# Comment on Chwalik-Pilszyk et al. Application of Polyurethane Foam as a Material for Reducing Vibration of Wheelchair User. *Materials* 2025, *18*, 1280

**DOI:** 10.3390/ma18225132

**Published:** 2025-11-12

**Authors:** Mario Bernardo-Filho, Anelise Sonza, Ana Carolina Coelho-Oliveira, Danúbia da Cunha de Sá-Caputo, Redha Taiar

**Affiliations:** 1 Laboratório de Vibrações Mecânicas e Práticas Integrativas, Instituto de Biologia Roberto Alcantara Gomes, Universidade do Estado do Rio de Janeiro, Rio de Janeiro 20950-003, RJ, Brazil; bernardofilhom@gmail.com (M.B.-F.); anacarol_coelho@hotmail.com (A.C.C.-O.); dradanubia@gmail.com (D.d.C.d.S.-C.); 2 Programa de Pós-Graduação em Ciências do Movimento Humano, Departamento de Fisioterapia, Centro de Ciências da Saúde e do Esporte, Universidade do Estado de Santa Catarina, Florianópolis 88080-350, SC, Brazil; anelise.sonza@gmail.com; 3 Department of Sport Sciences, Université de Remis Champagne-Ardenne, MATériaux et Ingénierie Mécanique (MATIM-UR 3689), 51687 Reims Cedex 2, France

First of all, we would like to congratulate the authors [1], who published the interesting paper entitled “Application of Polyurethane Foam as a Material for Reducing Vibration of Wheelchair User” in the journal *Materials* (Basel). This study aimed to design and test the effectiveness of utilizing a polyurethane cushion to reduce the whole-body vibration (WBV) acting on a person while moving in a wheelchair.

Although it was not clear if the model was validated, the study concluded that the presented model could become a fascinating tool for future analysis of mechanical vibrations of people with different mobility statuses, moving passively on several types of wheelchairs on surfaces whose irregularities can be accounted for by an appropriate form of kinematic excitation. The approach used in this study is likely to be helpful in selecting a wheelchair and seat cushion to counteract and minimize the mechanical vibrations perceived by humans.

The relevant study opens an extraordinary opportunity to discuss the effects of the mechanical vibrations used in systemic vibratory therapy (SVT). In SVT, mechanical vibrations are transmitted to the body of an individual that is in contact with the base of a vibrating platform producing the “whole-body vibration exercise”. The protocols of SVT use mechanical vibration with biomechanical parameters and other very well defined parameters, as it is indicated in recent guidelines [2]. Moreover, various clinical applications of SVT, in populations of different ages, have been reported. These applications are related to several biological effects, such as an increase in muscular strength, a decrease in pain, improvement in quality of life, flexibility, and functional capability [3,4]. Also, mechanical vibrations are relevant for piezoelectric stimulation for bone mineral density increase [5], which can be of particular interest for wheelchair users.

The mechanical vibrations acting on a person while moving in a wheelchair may not be as harmful as previously thought. The biomechanics of the sitting position itself started to be studied at the end of the XIX century [6] and continues at present [7]. The long exposition to the sitting position, with or without vibration, shows its impact on increased higher disc pressures compared to standing or lying positions [8] and classical electromyography studies from the 1970s in several contexts demonstrate a similar result [9,10,11,12,13]. Nevertheless, considering subjects’ comfort ratings, the theoretical ideal sitting position is not the preferred position [14]; it can be speculated and observed in clinics that for wheelchair users, the ideal sitting posture is not followed since other orthopedic compensations develop simultaneously. In this case, should the polyurethane cushion be used to improve sitting posture or focus mainly on decreasing whole-body vibrations? First, it would be interesting to try to verify the effects of the whole-body vibration effect due to the movement of a wheelchair, mainly because WBV exposition in wheelchair users has yet to be compared to car driver’s exposition. Moreover, considering the findings described by [1], the possibility of adjusting the polyurethane cushion to direct the transmission of mechanical vibration with defined biomechanical parameters specific to the individual, in this case to wheelchair users, would be valuable. How can polyurethane be optimized for mechanical vibration in order to realize its potential to help and improve the quality of life of the individual who is sitting in a wheelchair?

Putting together all the previous concepts in the current comment, it would be relevant to emphasize the dual function of the polyurethane cushion, not only as a passive vibration-dampening element but also as a potentially active medium for delivering controlled biomechanical stimulation. These insights contribute to an innovative perspective and approach that could lead to novel therapeutic strategies, mainly if the cushion’s physical properties could be fine-tuned to achieve specific and controlled mechanical vibrational profiles [15]. Moreover, the discussion of the current comment seems to align with recent research trends focusing on the therapeutic use of mechanical stimuli in rehabilitation settings.
